# Study of Absorption Characteristics of the Total Saponins from Radix *Ilicis Pubescentis* in an In Situ Single-Pass Intestinal Perfusion (SPIP) Rat Model by Using Ultra Performance Liquid Chromatography (UPLC)

**DOI:** 10.3390/molecules22111867

**Published:** 2017-11-01

**Authors:** Guojun Kuang, Huan Yi, Mingjuan Zhu, Jie Zhou, Xueying Shang, Zhongxiang Zhao, Chenchen Zhu, Qiongfeng Liao, Shixia Guan, Lei Zhang

**Affiliations:** 1 School of Chinese Materia Medica, Guangzhou University of Traditional Chinese Medicine, Guangzhou 510006, China; kenbenkenben@163.com (G.K.); yi317296805@163.com (H.Y.); zhumingjuan123@163.com (M.Z.); zhoujie_gz@163.com (J.Z.); 13430286922@163.com (X.S.); zzx37@163.com (Z.Z.); zhuchenchen@vip.sina.com (C.Z.); liaoqf2075@yahoo.com (Q.L.); drguan@163.com (S.G.); 2 Division of Biochemical Drugs, Guangzhou Institute for Drug Control, Guangzhou 510160, China

**Keywords:** intestinal permeability, in situ single-pass intestinal perfusion, total saponins, Radix *Ilicis Pubescentis*, oral drug absorption, UPLC

## Abstract

In contrast to the extensively reported therapeutic activities, far less attention has been paid to the intestinal absorption of the total saponins from Radix *Ilicis Pubescentis* (in Chinese Mao-Dong-Qing, MDQ). This study aimed to investigate the intestinal absorption characteristics of ilexgenin A (C1), ilexsaponin A1 (C2), ilexsaponin B1 (C3), ilexsaponin B2 (C4), ilexsaponin B3 (DC1), and ilexoside O (DC2) when administrated with the total saponins from MDQ (MDQ-TS). An UPLC method for simultaneous determination of C1, C2, C3, C4, DC1, and DC2 in intestinal outflow perfusate was developed and validated. The absorption characteristics of MDQ-TS were investigated by evaluating the effects of intestinal segments, drug concentration, P-glycoprotein (P-gp) inhibitor (verapomil), endocytosis inhibitor (amantadine) and ethylene diamine tetraacetic acid (EDTA, tight junction modulator) on the intestinal transportation of MDQ-TS by using a single-pass intestinal perfusion (SPIP) rat model, and the influence of co-existing components on the intestinal transport of the six saponins was discussed. The results showed that effective apparent permeability (P_app_) of C1, C2, C3, C4, and DC2 administrated in MDQ-TS form had no segment-dependent changes at low and middle dosage levels. C1, C2, C3, D4, DC1, and DC2 administrated in MDQ-TS form all exhibited excellent transmembrane permeability with P_app_ > 0.12 × 10^−2^ cm·min^−1^. Meanwhile, P_app_ and effective absorption rate constant (K_a_) values for the most saponins showed concentration dependence and saturation characteristics. After combining with P-gp inhibitor of verapamil, P_app_ of C2, C3, and DC1 in MDQ-TS group was significantly increased up to about 2.3-fold, 1.4-fold, and 3.4-fold, respectively in comparison to that of non-verapamil added group. Verapamil was found to improve the absorption of C2, C3, and DC1, indicating the involvement of an active transport mechanism in the absorption process. Compared with the non-amantadine added group, the absorption of C1, C2, C4, DC1, and DC2 were decreased by 40%, 71%, 31%, 53%, and 100%, respectively. P_app_ for the six target compounds increased up to about 1.2–2.1-fold in comparison with the non-EDTA added, respectively. The gastrointestinal transport of MDQ-TS could be greatly promoted by EDTA, and inhibited by amantadine, implying that the intestinal absorption of MDQ-TS was by passive diffusion and endocytosis process. Compared with monomer administration group, the intestinal absorption of C3, C4, DC1, and DC2 was significantly improved by co-existing components in MDQ-TS, and the non-absorbable saponins of C4, DC1, and DC2 unexpectedly showed sufficient intestinal permeability with P_app_ > 0.12 × 10^−2^ cm·min^−1^. This suggested that compounds orally administrated in TCM extract forms displayed unique intestinal absorption characteristics different from those of monomers, and the enhancing intestinal absorption of MDQ-TS reflected a holistic and specific view of traditional Chinese medicines (TCMs).

## 1. Introduction

**Figure 1:**
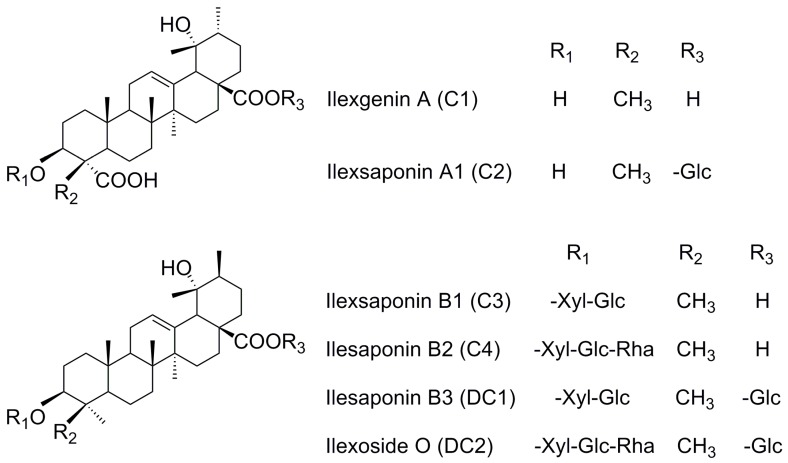
Chemical structures of IlexgeninA (C1), Ilexsaponin A1 (C2), Ilexsaponin B1 (C3), Ilesaponin B2 (C4), Ilesaponin B3 (DC1) and Ilexoside O (DC2).

Many different classes of natural products, including steroids, triterpenoids, lignins, and phenols, have been isolated from Radix *Ilicis Pubescentis* (Mao-Dong-Qing in Chinese, MDQ), the dried roots of *Ilex pubescens* Hook et Arn. (Aquifoliaceae). Radix *Ilicis Pubescentis* is widely distributed in Southern China [1,2,3], and are known for their medicinal properties that help in treating cardiocerebral, vascular, and arterial thrombotic diseases such as stroke, coronary arterial thrombosis, thromboangiitis obliterans, hyperlipidemia, and thrombophlebitis [4,5,6,7]. In addition, the plant has been used for alleviating upper respiratory infections and other inflammatory diseases [8]. It has been used as main ingredient in many formulae, such as Mao-Dong-Qing capsules, a compound in hairy holly and aluminum clofibrate tablets, Xue-Shuan-Xin-Mai-Ning tablets, and Mai-Kui-Kang aerosol. According to literature, triterpenoids are considered as the dominant active components, and more than 40 individual pentacyclic triterpenoids have been identified in Radix *Ilicis Pubescentis*, which possess the bioactivities of inhibiting platelet aggregation, preventing thrombosis, enlarging blood vessels, improving microcirluation, reducing cardiacischemia, and enhancing anoxia resistance [3,9,10]. Ilexgenin A (C1), one of the major components of MDQ, as well as a sapogenin, has been extensively investigated. In an in vivo study, C1 was found to be the main metabolite of ilexsaponin A1 (C2) following biotransformation by intestinal flora [11]. However, neither the parent drug nor the metabolites were detected in the rat plasma by LC-UV or LC-ELSD (evaporative light scattering detection) techniques when C2 or C1 was administered orally. Liu et al. analyzed plasma concentrations following the oral administration of C1, and found the maximum concentration was approximately 350 ng·mL^−1^, even after administration of a large dose (100 mg/kg), which indicated poor oral bioavailability [12]. Due to a lack of reference substances, little is known about the in vivo features of the other saponins in MDQ. The compounds ilexsaponin A1 (C2), ilexsaponin B1 (C3), ilexsaponin B2 (C4), ilexsaponin B3 (DC1), and ilexoside O (DC2), which contains a similar aglycon as C1, were separated in our laboratory. Pentacyclic triterpenoid saponins are universally considered to be a class of ingredients with poor oral bioavailability owing to high polarity, poor lipophilicity, high molecular weight, and poor membrane permeability. However, this information has been only inferred from compound structure or the properties of a small number of analogues. Such knowledge is insufficient to permit a deep understanding of the in vivo process for this kind of natural material. In our preliminary study, we found that C1, C2, C3, C4, DC1, and DC2 (Figure 1) possessed a suitable lipophilic property as far as druggability was concerned, with oil-water partition coefficient (K_ow_) values from −1 to 2. Currently, because very few systematic studies have been conducted on the intestinal absorption and transport mechanism of the triterpenoid saponins in MDQ, the precise explanation for low oral bioavailability is unknown. It is well known that screening of the drugs for the absorption characteristics is imperative to select right candidate and to prevent the termination of the developing candidate at the later stage of drug development program. Studies on the absorption and transport mechanism of drugs can also provide references for formulation development, prescription, and preparation technology optimization, clinical rational drug use, and so on. Thus, more experiments and studies need to be conducted.

Oral administration is the preferred path for the delivery of most drugs into the body. Before reaching the bloodstream, drugs administered orally are usually absorbed from the small intestine. Among the permeability models available to study transepithelial transport, the in situ intestinal perfusion technique is the preferred approach when bio-relevance and accurate prediction, rather than high-throughput screening are favored [13]. This approach provides an intact blood supply and a functional intestinal barrier, and allows study of the influence of transporters on absorption [14,15]. In this paper, the absorption and transport mechanism of the saponins in MDQ in the small intestine of rats was investigated using an in situ single-pass perfusion model combined with an efficient quantitation method using UPLC.

## 2. Results

### 2.1. Method Validation

**Figure 2:**
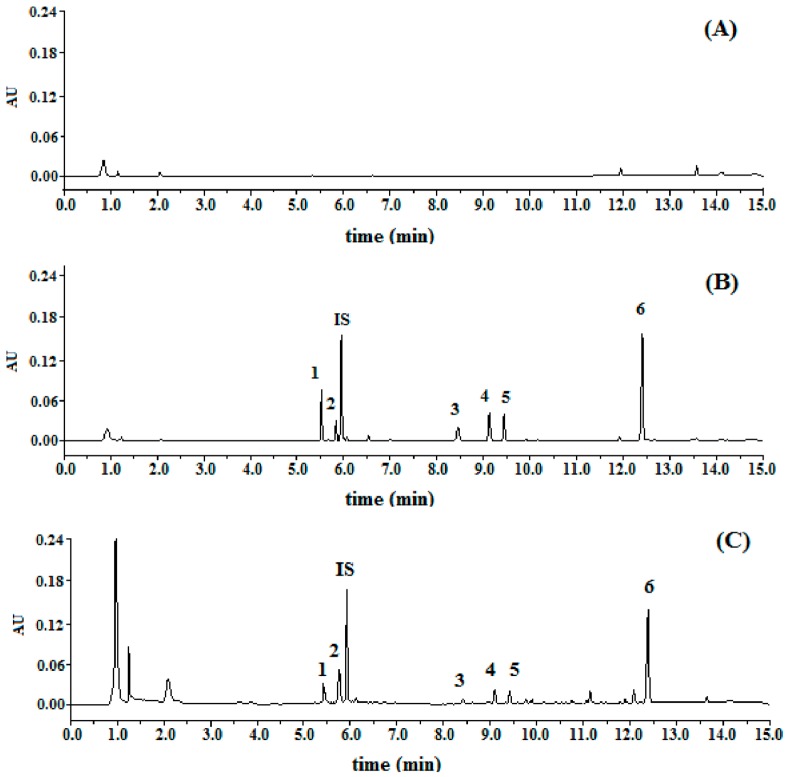
The representative UPLC-UV chromatograms. (**A**) Blank outflow of intestinal perfusate; (**B**) intestinal perfusate matrix spiked with C1, C2, C3, C4, DC1, DC2, and IS; (**C**) the intestinal outflow perfusate collected at 20–30 min after in situ single-pass perfusion with MDQ-TS contained K-R buffer. The potential interference of intestinal perfusate endogenous with the analytes was assessed by comparing the chromatograms of the above processed intestinal perfusion samples.

Specificity: Typical chromatograms were presented in Figure 2. The relative retention time of DC2, DC1, C4, C2, C3, and C1 were 0.93, 0.98, 1.42, 1.54, 1.59, and 2.09, respectively, by taking digoxin as (internal standard, IS). All the peaks of the analytes and IS were detected with excellent resolution, as well as peak shapes, and no interference from the endogenous substances was observed. The analytes could be easily differentiated from the biological matrix and quantitatively determined.

Calibration curve and limit of quantification (LOQ): The calibration curves showed good linearity over the concentration range of 20–1000 µg·mL^−1^ for C1, and 8.0–400 µg·mL^−1^ for C2, C3, C4, DC1, and DC2. The typical calibration plot equations and their correlation coefficients were calculated as follows: C1, y = 3.250x + 0.0135 (r = 0.9993); C2, y = 2.252x + 0.0036 (r = 0.9992); C3, y = 1.858x + 0.0032 (r = 0.9994); C4, y = 1.451x + 0.0019 (r = 0.9992); DC1, y = 1.060x + 0.0018 (r = 0.9993); DC2, y = 2.577x + 0.0039 (r = 0.9993). LOQ was found to be 480.5 ng·mL^−1^ for C1, 690.8 ng·mL^−1^ for C2, 856.4 ng·mL^−1^ for C3, 1.14 µg·mL^−1^ for DC1, and 636.0 ng·mL^−1^ for DC2.

Accuracy and precision: The accuracy ranged from −3.5~1.6% for C1, −4.3% to –2.9% for C2, −2.9~6.3% for C3, 2.6~5.1% for C4, −2.6~4.9% for DC1 and −3.7~4.5% for DC2. The intra-day and inter-day precision for C1, C2, C3, C4, DC1, and DC2 was less than 4.6%, 4.9%, 4.1%, 5.2%, 5.4%, and 4.8%, respectively. The results indicated that overall reproducibility of the method was acceptable.

Extraction recovery and matrix effect: The mean extraction recoveries determined using six replicates of quality control (QC) samples at three concentration levels were found to be 94.2 ± 3.0% for C1, 97.8 ± 2.4% for C2, 94.3 ± 2.0% for C3, 105.7 ± 4.1% for C4, 92.4 ± 4.8% for DC1, 95.3 ± 1.2% for DC2, and 102.1 ± 3.3% for IS. As for matrix effect, the average matrix effect values of the analytes at three concentration levels ranged from 98.9% to 102.1% for C1, 97.6% to 104.9% for C2, 104.1% to 108.3% for C3, 101.3% to 103.7% for C4, 99.3% to 105.6% for DC1, and 93.7% to 98.5% for DC2. The results indicated that the method was free from matrix effect.

Stability: C1, C2, C3, C4, DC1, and DC2 were proved to be stable for at least 12 h in outflow perfusate matrix at 37 °C, for 24 h in autosampler condition after preparation, for 14 days under cold storage and within three freeze-thaw cycles with a concentration bias at three QC levels (RE) ranging from −6.7% to 4.2%.

### 2.2. Absorption of MDQ-TS and Six Monomers in Different Intestinal Segments

The absorption parameters of the six saponin monomers and MDQ-TS are listed in Table 1 and Table 2. It was demonstrated that the order of P_app_ for C1, C2, and C3 monomers was duodenum ≈ jejunum ≈ ileum, and the same as the order of K_a_ for the three analytes monomers. The results (Table 1) indicated that the intestinal absorption of C1, C2, and C3 monomers was not segmental dependent. For SPIP techniques, drugs with P_app_ > 0.12 × 10^−2^ cm·min^−1^ in the rat small intestine are classified as completely absorbed (highly permeable) group [16]. For C1, C2, and C3 monomers, the P_app,rat_ in duodenum/jejunum/ileum was found to be 0.33/0.35/0.30 × 10^−2^ cm·min^−1^, 0.24 /0.25 /0.25 × 10^−2^ cm·min^−1^ and 0.23 /0.23 /0.21 × 10^−2^ cm·min^−1^, respectively. These results suggested that C1, C2, and C3 were highly permeable substances. On the contrary, C4, DC1, and DC2 monomers could not be absorbed by small intestine at all. As for MDQ-TS administration group (Table 2), the P_app,rat_ of C1, C2, C3, C4, and DC2 had no obvious differences among duodenum, jejunum, and ileum at low and middle concentration levels. Nevertheless, the orders of P_app_ for the six analytes in rats perfused with MDQ-TS at high concentration level (5.0 mg·mL^−1^) appeared the following subsequence: duodenum ≈ jejunum > ileum (*p* < 0.05 for C1, C3, C4, and DC1).

### 2.3. Absorption of MDQ-TS at Different Concentrations

In terms of MDQ-TS perfusion study, Table 2 showed that except DC2 in 1.25 mg·mL^−1^ of MDQ-TS group, C1, C2, C3, D4, DC1, and DC2 all exhibited excellent transmembrane permeability with P_app_ > 0.12 × 10^−2^ cm·min^−1^. Meanwhile, P_app_ and K_a_ for the most saponins showed a concentration-dependent trend in the jejunum, ileum, and duodenum. Briefly, in segments of jejunum and duodenum, the P_app_ value of C1, C2, C3, C4, DC1, and DC2 increased and tended to balance with the increasing concentration within the range of 1.25–5.0 mg·mL^−1^. In segment of ileum, the P_app_ values for most analytes first increased and then decreased with the increasing concentration. The results indicated that there might be active transportation process in the absorption mechanism of the six analytes when administered in MDQ-TS form.

### 2.4. Effects of Absorption Enhancers and P-glycoprotein (P-gp) Inhibitors on the Intestinal Absorption of MDQ-TS

**Figure 3:**
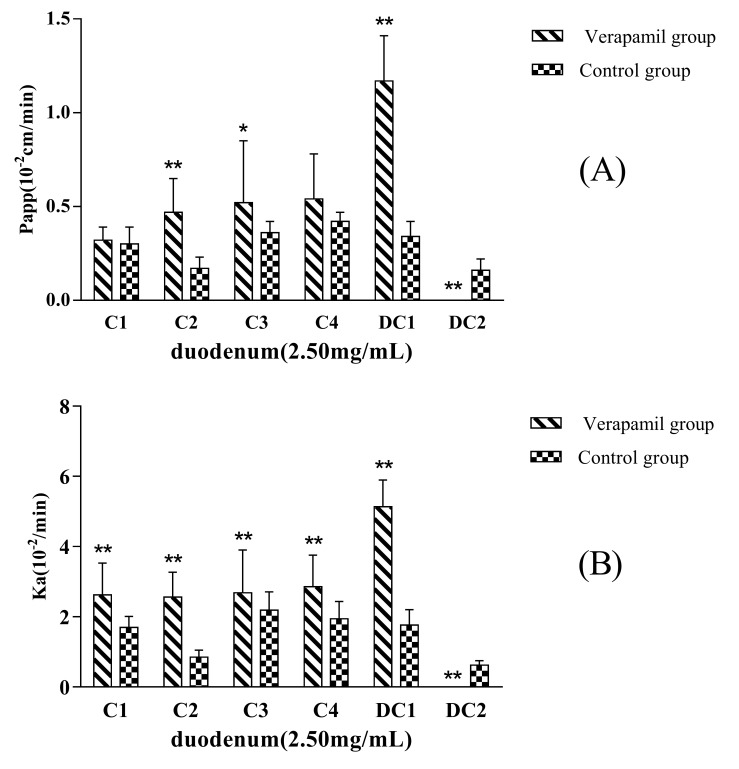
P_app_ (**A**) and K_a_ (**B**) of the six analytes in duodenum obtained after in situ single-pass perfusion of MDQ-TS (2.5 mg/mL) with or without verapamil. The rat duodenum (~10 cm) was used to evaluate the intestinal permeability of MDQ-TS. Data was expressed as mean ± SD of five independent experiments each group. * *p* < 0.05 versus non-verapamil group; ** *p* < 0.01 versus non-verapamil group.

The P_app_ and K_a_ values for MDQ-TS in the presence or absence of P-gp inhibitor, 200 μmol·L^−1^ of Verapamil, had been showed in Figure 3. The P_app_ of C2, C3, and DC1 in rats increased up to about 2.3-fold, 1.4-fold, and 3.4-flod in comparison with the non-Verapamil added group (control group), respectively. The Ka of C1, C2, C3, C4, and DC1 increased up to about 1.5-fold, 4.2-fold, 1.2-fold, 1.5-fold, and 2.9-fold in comparison with the non-Verapamil added, respectively. These results suggested that the inhibition of the intestinal P-gp by Verapamil could significantly enhance the permeability of some saponins in MDQ-TS. DC2 in non-Verapamil group was characteristic of high permeability, while it could not be absorbed by the small intestine at all in Verapamil added group. Unfortunately, the mechanism of Verapamil’s inhibition effect on intestinal absorption of DC2 has not been illustrated in the present study.

**Figure 4:**
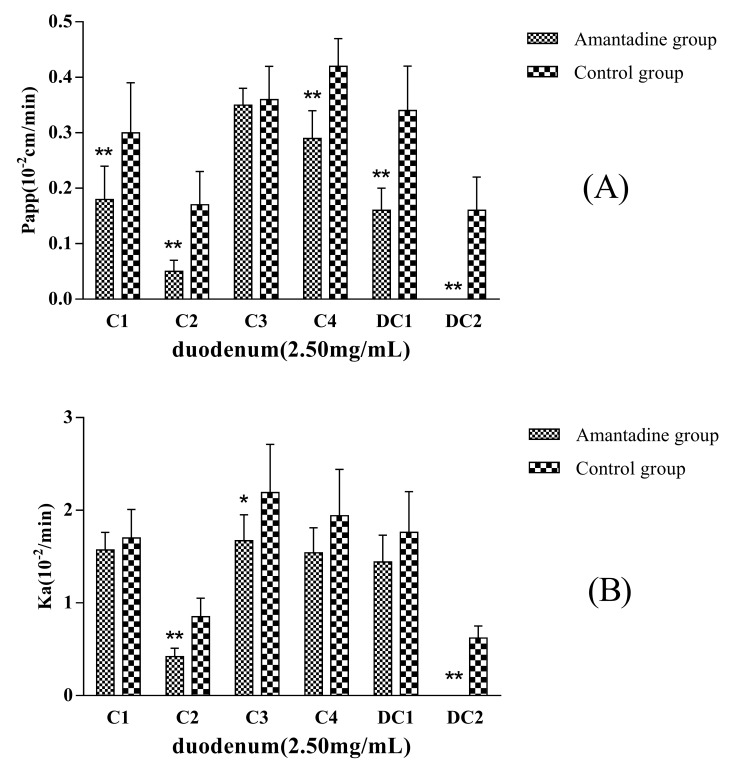
P_app_ (**A**) and K_a_ (**B**) of the six analytes in duodenum obtained after in situ single-pass perfusion of MDQ-TS (2.5 mg/mL) with or without amantadine. The rat duodenum (~10 cm) was used to evaluate the intestinal permeability of MDQ-TS. Data was expressed as mean ± SD of five independent experiments each group. * *p* < 0.05 versus non-amantadine group; ** *p* < 0.01 versus non-amantadine group.

Amantadine (2.5 mmol·L^−1^) was added to the inflow perfusate as an endocytosis inhibitor to evaluate whether pinocytosis was involved in the MDQ-TS transmembrane transport process. Compared with the non-Amantadine added group (control group), the P_app_ and K_a_ values of C1, C2, C4, DC1, and DC2 showed significantly decreasing trend (Figure 4), in particular, the absorption of DC2 was completely inhibited when co-perfusion with Amantadine. The absorption of C1, C2, C4, DC1, and DC2 were decreased by 40%, 71%, 31%, 53%, and 100%, respectively. The results were of great significant (*p* < 0.01) compared with non-Amantadine added group. Therefore, it was inferred that endocytosis effects should be involved in the intestinal transportation process of the five saponins.

**Figure 5:**
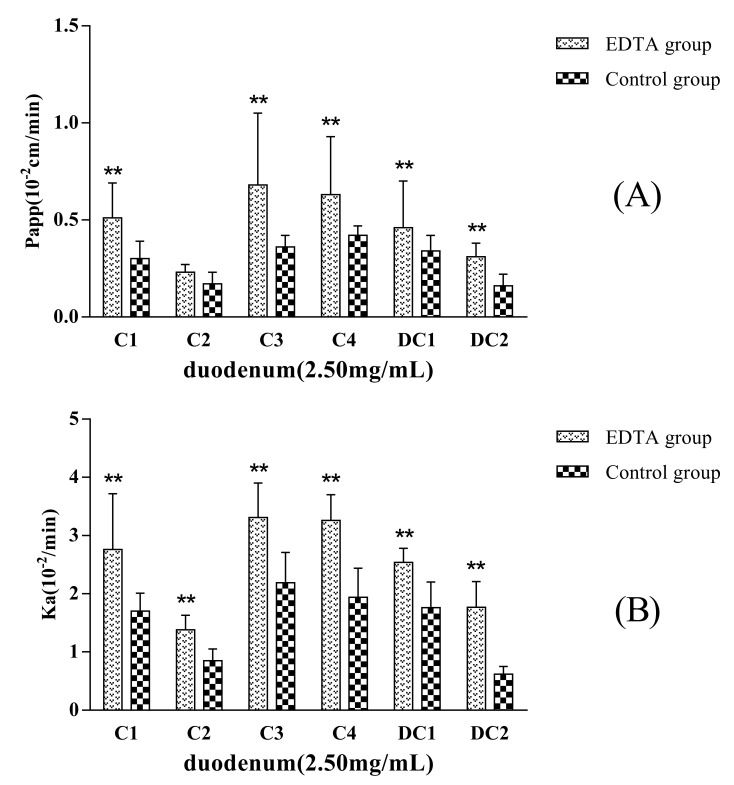
P_app_ (**A**) and K_a_ (**B**) of the six analytes in duodenum obtained after in situ single-pass perfusion of MDQ-TS (2.5 mg/mL) with or without EDTA. The rat duodenum (~10 cm) was used to evaluate the intestinal permeability of MDQ-TS. Data was expressed as mean ± SD of five independent experiments each group. ** *p* < 0.01 versus non-EDTA group.

EDTA, a kind of metal chelator, can destroy the intercellular structure that is named tight junction of the epithelia and increase the paracellular permeation of hydrophilic macromolecules. If the transport mechanism of a drug involves passive diffusion, its transport will be improved when epithelial tight junction structure is opened or destroyed. Figure 5 showed that the permeability of C1, C2, C3, C4, DC1, and DC2 administrated in MDQ-TS form increased when a concentration of 2.5 mmol·L^−1^ EDTA was added in the inflow perfusate. P_app_ (and K_a_) values for C1, C2, C3, C4, DC1, and DC2 increased up to about 1.7-fold (1.6-), 1.2-fold (1.6-), 1.9-fold (1.5-), 1.5-fold (1.5-), 1.4-fold (1.4-), and 2.1-fold (3.0-) in comparison with the non-EDTA added (control group), respectively. Thus, these results indicated that the mechanism of intestinal absorption mechanism of MDQ-TS involved passive transcellular diffusion. Their oral bioavailability could be improved by changing the rheological properties of intestinal mucus.

### 2.5. Comparison of the Absorption Behavior of MDQ-TS and Monomers

**Figure 6:**
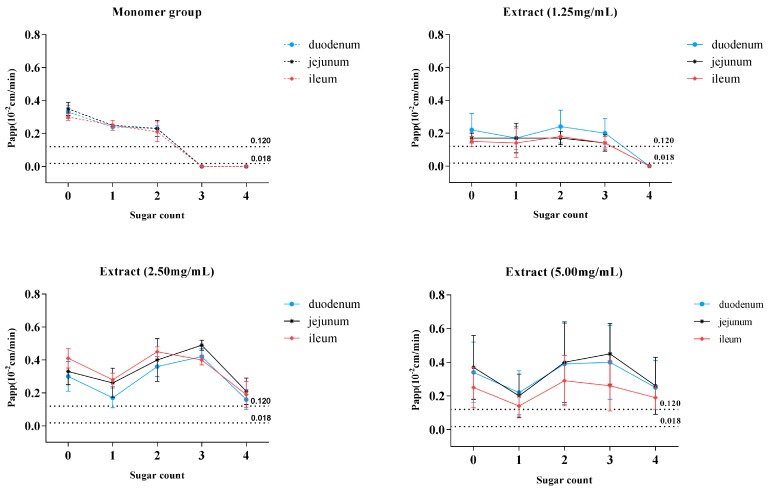
The relationship of saponin glycoside numbers with P_app_ value in monomer groups and different concentration of MDQ-TS groups. (**A**) Monomers groups; (**B**) 1.25 mg·mL^−1^ of MDQ-TS group; (**C**) 2.5 mg·mL^−1^ of MDQ-TS group; and (**D**) 5.0 mg·mL^−1^ of MDQ-TS group. By plotting the glycoside numbers of C1, C2, C3, C4, DC1, and DC2 versus their P_app_ values, the relationships between glycoside numbers and intestinal permeability of these saponins were clearly seen. Meanwhile, the interaction of co-existing components in MDQ-TS on the intestinal transportation of these saponins could be observed by comparing between saponin monomers groups and different concentration of MDQ-TS groups. P_app_ > 0.12 × 10^−2^ cm·min^−1^ are classified as the completely absorbed (highly permeable) group, and P_app_ < 0.018 × 10^−2^ cm·min^−1^ as the poorly absorbed group.

Drugs were divided into four categories (Class I to Class IV) following FDA guidance for the industry, based on the permeability and solubility of the compounds (Biopharmaceutics Classification System, BCS) [14]. As judged from Table 1 and solubility of the six saponins (data not shown), C1, C2, and C3 monomers were categorized into class II (low-solubility, high-permeability), which indicated that these compounds might develop into oral formulation if the necessary pharmaceutical means for improving the dissolution of these compounds were applied. The placement of C4, DC1, and DC2 monomers in class IV (low-solubility, low-permeability) indicated that these saponins were not suitable for oral administration without considering the role of intestinal flora. We observed that the six target saponins had the similar aglycone structures, while K_a_ and P_app_ values showed a downward trend with an increasing number of glycosides (Figure 6A). The data in Table 1 and Figure 6A showed that when the six saponins were administered in monomer forms, C1, C2, and C3, being characteristic of glycoside numbers less than three, showed excellent intestinal permeability with P_app_ > 0.12 × 10^−2^ cm·min^−1^, while no absorption was observed in terms of C4, DC1, and DC2 (glycoside numbers more than three). The nonabsorbent feature of C4, DC1, and DC2 could be explained by the “rule of five,” i.e., either because of large molecular mass or excess hydrogen bond numbers [17]. Surprisingly, the non-absorbent compounds of C4, DC1, and DC2 could even be absorbed by the small intestine if administrated in MDQ-TS form (Figure 6B–D), with the P_app_ values of C4, DC1, and DC2 increasing to above 0.12 × 10^−2^ cm·min^−1^, which indicated a significant improvement for the intestinal absorption of these saponins by the co-existing components in MDQ-TS.

### 2.6. Apparent Oil-Water Partition Coefficient

**Figure 7:**
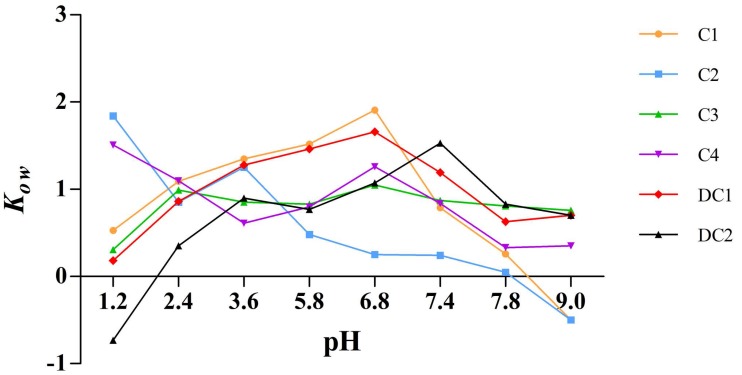
K_ow_–pH profiles of C1, C2, C3, C4, DC1, and DC2. The equilibrium partition of the six saponins monomers between *n*-octanol and different pH values of phosphate buffer (pH 1.2, 2.0, 3.6, 5.8, 6.8, 7.4, 7.8, and 9.0) was determined by the traditional shake-flask method coupled with validated HPLC quantification methods.

The apparent oil-water partition coefficient, K_ow_, is an important property reflecting the membrane transport of drugs. Figure 7 illustrated that the lipophilicity of C1, C2, C3, C4, DC1, and DC2, which is expressed by K_ow_, was within the range of −1 to 2. The K_ow_ of the six saponins indicated excellent liposolubility as far as druggability was concerned and, thus, we deduced that the poor oral bioavailability of these components should not be ascribed to the lipophilicity [18].

## 3. Discussion

In this paper, we focused on the six active compounds in MDQ-TS with similar aglycone-based structures, but differing numbers of glycoside groups, by using an in situ single-pass perfusion method. Since no studies on the intestinal absorption of C1, C2, C3, C4, DC1, and DC2 administered in MDQ-TS forming simultaneously can be found, it is suggested that this research would be the first to focus on the intestinal absorption of active compounds in MDQ-TS.

Generally, there are three absorption models containing the rat in situ single-pass perfusion system, the rat everted gut sac method and the monolayer of human colon adenocarcinoma cells in line Caco-2 cell model. In situ SPIP technique provides several advantages over the other methods for the determination of effective permeability. This technique provides conditions closer to what is faced following the oral administration, preserved microclimatic condition, and is less sensitive to pH variations. It provides the unique advantages of the experimental control and the ability to study regional differences, factors that may influence the intestinal absorption of the compound. It has an advantage that transporter activity also gets counted in the same experimental condition, which will also help to predict accurate human intestinal permeability and classify the drug candidate according to BCS [19,20,21]. Hence, in this study the rat in situ SPIP model was used to investigate the intestinal absorption characteristics of C1, C2, C3, C4, DC1, and DC2, a group of pentacyclic triterpenoid saponins.

In the study, we systematically characterized the effect of the concentration, P-gp inhibitors, endocytosis inhibitors, as well as paracellular pathway enhancers on the intestinal absorption of MDQ-TS. The results illustrated that the P_app_ value of the six saponins increased and tended to balance with the increasing concentration (concentration-dependent and saturation phenomena). It suggested that the transport mechanism of these compounds might fit the carrier-mediated transport mechanism and/or endocytosis pathway if administrated in MDQ-TS form. To further verify the effect of transporters on the absorption of MDQ-TS, the effect of P-gp inhibitor, endocytosis inhibitor, and paracellular pathway enhancer were studied. P-gp was discovered over forty years ago by Juliano and Ling as a highly-abundant, high molecular weight, integral plasma membrane glycoprotein in drug-selected Chinese hamster ovary cells [22]. It has been proved that P-gp confers multidrug resistance (MDR) by acting as an energy-dependent drug efflux pump [23]. As for P-gp, one of the most interesting and controversial issues surrounding P-gp concerns the mechanism whereby a single transporter molecule can reduce the intracellular concentration of a large number of structural heterogeneous substrates. Unfortunately, the actual mechanism of drug transport by P-gp is not well understood at present, and one of the most intriguing models to account for the interaction of P-gp with drugs is that P-gp acts as a “hydrophobic vacuum cleaner, HVC” [24].

The P-gp are generally located specifically in the apical (intestinal luminal side) or basolateral (blood/plasma side) membranes of the enterocytes. This may drive compounds out of cell or pump a component from the subserosal back into the submucosa of the intestinal lumen, preventing their absorption into blood. Thus suppression of P-gp expression will increase the intestinal absorption of P-gp-mediated drugs. Verapamil was used to judge whether the intestinal absorption of MDQ-TS would be affected by P-gp preliminarily. The P_app_ of the three saponins increased when verapamil was added to the medium containing 2.5 mg·mL^−1^ of MDQ-TS. The results showed that the absorption of MDQ-TS in the intestine might be by active transportation mediated by the P-glycoprotein transporter. In endocytosis, the characteristic of intake of the drug inside cell, or release from intracellular to extracellular areas, occurs through active deformation of the cell membranes. It is particularly important for the absorption of protein, peptide, fat-soluble vitamins, and triglycerides, but generally insignificant to most small-molecule drugs. Except for C1, the other five compounds containing the similar aglycon as C1 also contain a monosaccharide (C2), disaccharide (C3), trisaccharide (C4, DC1), and tetrasccharide (DC2) in the structure. Although the five triterpenoid saponins possess significant differences in polarity, molecular size, solubility, and liposolubility, endocytosis effect was involved in the intestinal transportation for all five saponins. More research is needed to determine whether the effects are universal to most kinds of pentacyclic triterpenoids when administrated in TCM formulation. Passive transport refers to drugs that are transported from the high-concentration side to the low-concentration side, along with the concentration gradient diffusion principle. The tight junction degree of the intestinal epithelial cells is one of the important influential factors on the intestinal absorption of drugs mediated by passive transport ways. EDTA can enhance the paracellular permeability of intestinal peptide drugs by opening epithelial tight junctions. The results of the effluence of EDTA on intestinal transport of MDQ-TS showed that the permeability of C1, C3, C4, DC1, and DC2 significantly increased when EDTA were added. These results further indicated that the intestinal absorption mechanism of most tested pentacyclic triterpenoids in MDQ-TS involved passive transcellular diffusion [25]. Based on the above results of this paper, we concluded that the intestinal absorption of most tested triterpenoid saponins in MDQ-TS was by passive diffusion and endocytosis as the domination process. Both paracellular and transcellular transport were observed to contribute to the drug intestinal absorption during the perfusion. Unfortunately, by now, due to a lack of reference substances, we did not achieve in-depth discovery on the intestinal transportation characteristics of these saponin monomers, which needs to be further investigated by the Caco-2 model.

Numerous studies have proven that administration of a natural material in a TCM extract formulation results in higher plasma concentration and oral bioavailability than when administered as a monomer [11,25,26,27,28,29]. Consequently, this feature of TCMs can explain why some categories of ingredients, such as flavonoids or pentacyclic triterpenoid saponins, have good efficacy and broad clinical applications despite unsatisfactory pharmaceutical properties, i.e., poor solubility, poor stability, and inappropriate lipid solubility. As is commonly found in many natural products, the uptake of the most components tested was significantly altered by administration in MDQ-TS form. Intestine permeability and absorption of C3, C4, DC1, and DC2 were greatly improved by the contribution of other components in MDQ-TS. Moreover, most of the triterpenoid saponins can be transformed into sapogenin by intestinal flora after oral administration, and the metabolites of sapogenins often possess more excellent pharmaceutical properties, such as higher permeability, higher solubility, more suitable liposolubility, and so on, in comparison with their corresponding saponins [30,31]. Previous studies have illustrated that the intestinal flora metabolismis of some saponins into sapogenins could also be improved by co-existing components in the TCM extract or its compound preparations [32]. Intestinal flora metabolism and enhancement of absorption effects by co-existing components in MDQ may probably explain why the monomer compounds with low oral bioavailability and even unabsorbent characteristics, such as C4, DC1, and DC2, exhibited excellent pharmacological efficacy in vivo when administered in TCM extract form.

There is a consensus that poor oral bioavailability of most pentacyclic triterpenoid saponins arises from high polarity, unsuitable liposolubility, and low membrane permeability of these compounds [30,33]. However, the apparent oil-water partition coefficient test (Figure 7) showed that all of the six saponins had suitable liposolubility as far as druggability was concerned, with a K_ow_ value between −2 and 1, and C1, C2, and C3 monomers also exhibiting good membrane permeability. Previously, the absolute oral bioavailability of C1 could be only calculated based on literature data [12,34], which yielded a low value. The permeability of the six saponin monomers decreased with an increase in the number of glycoside groups (Figure 6), and pentacyclic triterpenoid saponins were investigated as a class of ingredients with poor oral bioavailability [34,35]. Considering the results of the in situ single-pass perfusion experiments, we deduced that membrane permeability and liposolubility should not be the major reasons responsible for the poor oral bioavailability of saponins in MDQ.

In conclusion, the intestinal absorption of C1, C2, and C3 monomers had no segment-dependent changes, and C4, DC1, and DC2 monomers could not be absorbed by the gastrointestinal tract at all. However, C1, C2, C3, D4, DC1, and DC2 administered in MDQ-TS form all exhibited excellent transmembrane permeability. The feature that co-existing ingredients contained in MDQ-TS significantly improved the intestinal absorption of triterpenoid saponins explain why MDQ had good efficacy and broad clinical applications despite unsatisfactory pharmaceutical properties of the components. Especially, the non-absorbent compounds of C4, DC1, and DC2 could, surprisingly, be absorbed by the small intestine if administered in MDQ-TS form. In contrast to the extensively-reported passive intestinal transportation of more than 90% triterpenoid saponins, both passive, endocytosis and P-gp-mediated active transport mechanisms were involved in the intestinal transportation of MDQ-TS. These suggested that compounds orally administered in TCM extract forms displayed the unique intestinal absorption characteristics different from those of monomers, and reflected a holistic and specific view of traditional Chinese medicines (TCMs).

## 4. Materials and Methods

### 4.1. Ethics Statement

Sixty-five male healthy Sprague-Dawley rats (180–220 g, eight-weeks-old) were obtained from the Experimental Animal Center of Guangzhou University of Traditional Chinese Medicine (Guangzhou, China). The animals were housed under standard conditions of light and dark cycles with food and water ad libitum. Animal experiments were carried out in strict accordance with the National Institute of Health Guidelines on the ethical use of animals, and the protocol was approved by the Animal Ethics Committee of Guangzhou University of Traditional Chinese Medicine (SCXK, licence no. 2008-0020). All surgeries were operated under urethane anesthesia, and every effort was made to minimize suffering.

### 4.2. Materials and Chemicals

Commercial herbal samples of Radix *Ilicis Pubescentis* (origin: Guangdong) processed by Zhixin Medicine Health Co., Ltd. (Guangzhou, China) was purchased from Da-Sen-Lin drug store in Guangzhou and authenticated by Prof. Jinsong Zhou (School of Chinese Materia Medica, Guangzhou University of Traditional Chinese Medicine, Guangdong, China) as the dried roots of *Ilex pubescens* Hook. et Arn. in Aquifoliaceae families. Reference standards (Figure 1) of ilexgenin A (C1), ilexsaponin A1 (C2), ilexsaponin B1 (C3), ilexsaponin B2 (C4), ilexsaponin B3 (DC1), and ilexoside O (DC2), the purity of which was proved to be above 98%, were isolated from Radix *Ilicis Pubescentis*, and contributed by Professor Zhongxiang Zhao. The structures of these self-made reference standards were fully characterized by chemical and spectroscopic methods [36,37,38,39,40]. Digoxin (batch number: 100015-200709) was purchased from the National Institute for the Control of Pharmaceutical and Biological Products (Beijing, China). HPLC-grade acetonitrile was obtained from Merck (Kga, Darmstadt, Germany). Deionized water used throughout the experiments was purified by a Millipore system (Millipore Co., Ltd., Bedford, MA, USA). Krebs-Ringer (K-R) solution (pH 7.4) was prepared based on report [14]. Other reagents were commercially available and were of analytically pure grade.

### 4.3. Preparation of Total Saponins of MDQ (MDQ-TS)

Dried and crushed plant material was macerated twice with distilled water (1:10, *w*/*v*) each time for 12 h. The aqueous solution was discarded, and the herbal powder was dried again in an oven with temperature set at 50 °C. The dried powder was accurately weighted and extracted twice with methanol (1:10, *w*/*v*) by sonication for 30 min at room temperature. The extracted solution was combined after filtration, and concentrated to a constant weight in a rotary vacuum evaporator at 60 °C (0.098 MPa). The obtained residual was crushed into powder. The total saponins’ purities and the content of C1, C2, C3, C4, DC1, and DC2 in final crushed powder (MDQ-TS) were determined to be 80.3%, 190.4 mg/g, 64.3 mg/g, 77.1 mg/g, 34.6 mg/g, 78.1 mg/g, and 57.5 mg/g, respectively, by our previously-established method [41].

### 4.4. Preparation of Perfusion Buffer Solutions

MDQ-TS perfusion samples at low, middle, and high concentration levels of 1.25, 2.5, and 5.0 mg·mL^−1^ were prepared by separately dissolving MDQ-TS in the minimum amount of DMSO and diluted with K-R buffer solution. The amount of DMSO was 0.5% in the final perfusion solutions. C1, C2, C3, C4, DC1, and DC2 perfusion samples with the concentration equivalent to the amount contained in 2.5 mg·mL^−1^ of MDQ-TS were individually prepared in the same way. Verapamil, amantadine and EDTA were directly dissolved in the MDQ-TS perfusate with terminal concentrations of 200 μmol·L^−1^, 2.5 mmol·L^−1^, and 2.5 mmol·L^−1^, accordingly, and used for investigating the mechanisms involved for absorption.

### 4.5. Instruments and Chromatographic Conditions

Liquid chromatographic separations of the analytes were performed using a Waters Acquity UPLC system combined (Milford, MA, USA) with a Waters Acquity HSS T3 UPLC column (2.1 × 100 mm, 1.8 μm) and photodiode array detector (PDA). The mobile phase consisted of A (acetonitrile) and B (0.05% aqueous phosphoric acid) using a gradient elution program of 25% A at 0–2 min, 25–38% A at 2–4 min, 38% A at 4–6.5 min, 38–65% A at 6.5–10 min, 65–85% A at 10–11 min, and 85% A at 11–13 min. The flow rate was set at 0.25 mL·min^−1^ with the column temperature maintained at 30 °C. The injection volume was 1 μL. The detection wavelength was set at 210 nm.

### 4.6. Pretreatment of Perfusate Samples

One hundred microliters of the outflow perfusate was successively spiked with 50 μL of digoxin solution (400 μg·mL^−1^ in methanol, IS) and 1.0 mL of acetonitrile, followed by vortex-mixing thoroughly for 3 min and centrifuging at 13,000× *g* for 5 min. One milliliter of supernatant liquor was collected and evaporated to dryness under a gentle stream of nitrogen on a 40 °C water bath. The residue was reconstituted with 200 μL of methanol, and then centrifuged again at 13,000× *g* for 3 min before injection into the UPLC system.

### 4.7. Method Validation

The chromatographic method was validated for specificity, linearity and LOQ, accuracy and precision, extraction recovery, matrix, and stability. The validation parameters were studied using drug-free (blank) intestinal perfusate mixture that had passed through intestinal segments, and had been collected from five different rats. Specificity was evaluated by analyzing the intestinal perfusion blank and perfusion blank spiked with analytes to investigate the potential interferences of intestinal perfusate endogenous with the analytes; calibration and quality control (QC) samples were obtained by spiking mixed working standard solution in blank intestinal perfusion buffer. Calibration curves were constructed in the range of 20–1000 μg·mL^−1^ for C1 (20, 40, 100, 200, 400, and 1000 μg·mL^−1^), and 8.0–400.0 μg·mL^−1^ for C2, C3, C4, DC1 and DC2 (8.0, 16.0, 40.0, 80.0, 160.0, and 400.0 μg·mL^−1^). A weighted (1/x^2^) linear least-squares linear regression method was used to determine the slope, intercept, and correlation coefficient; LOQ was determined by injection a series of standard solutions until the signal-to-noise (S/N) ratio was 10; Precision and accuracy were assessed at low, medium, and high concentration levels (40, 200, and 800 μg·mL^−1^ for C1; 16, 80, and 320 μg·mL^−1^ for C2, C3, C4, DC1, and DC2). Intra-day data was obtained by assaying six replicates at each concentration level in a day, and inter-day data was determined by analyzing QC samples in duplicates during three separate and successive days; The extraction recovery of the six analytes and IS from the outflow perfusate matrix was determined by comparing the mean peak areas of six replicates processed QC samples at each concentration level spiked before extraciton with those from outflow perfusate spiked after extraction; Matrix effect was assessed by comparing the mean peak areas of six replicates at each concentration level dissolved in the post-extracted intestinal perfusate matrix with those of neat standards (C1, C2, C3, C4, DC1, DC2, and IS) spiked in methanol at corresponding concentration levels; Stabilities of C1, C2, C3, C4, DC1, and DC2 in outflow perfusate matrix (12 h, 37 °C), processed samples (auto-sampler condition for 24 h), after freeze-thaw cycle and long-term cold storage (−20 °C, 14 days) were assayed by QC samples in six replicates at three concentration level.

### 4.8. In Situ Single-Pass Intestinal Perfusion Experiments

In situ single-pass intestinal perfusion (SPIP) studies were performed using an experimental design adapted from the literature with some modifications [42,43]. Briefly, male Sprague-Dawley rats weighing 180–220 g were acclimatized for at least one week before the experiment, and fasted for 18 h with free access to water before the perfusion experiment. Rats were anesthetized with an intraperitoneal injection of urethane (30 mg·kg^−1^ body weight), and kept on a homeothermic blanket to maintain normal body temperature. The abdomen was opened with a midline longitudinal incision, and three segments of rat intestine (duodenum, jejunum, and ileum) of approximately 10 cm each were carefully cannulated on two ends with plastic tubing. Initially, the perfusion solution free of drug (37 °C) was pumped by peristaltic pump (BT100-1F, Longer Pump Co., Baoding, China) through the cannulated segments at a flow rate of 1 mL/min for approximately 30 min in order to clean out any residual debris. Secondly, the perfusion solution containing a known concentration of substance (C1, C2, C3, C4, DC1, DC2, and MDQ-TS) was perfused at 37 °C through the intestinal lumen at a constant flow rate of 0.2 mL/min. The first 20 min of period corresponds to steady state, i.e., when the substance is in equilibrium with the intestinal membrane [21]. After steady state, samples were collected from distal portion of duodenum, jejunum and ileum at 10 min intervals (0–10, 10–20, 20–30, 30–40, 40–50, 50–60 min) in pre-weighted vials. Samples were immediately frozen at −20 °C until analysis by validated UPLC. During the experiment, care was taken to avoid disturbance of the circulatory system and the exposed segment was covered with a piece of sterilized gauze to keep it warm and moist by frequent application of warm (37 °C) saline. The intestinum tenue maintained its viability and intact state without disrupting the blood supply throughout the experimental period. Finally, animals were sacrificed after depth anesthesia by intraperitoneal injection of urethane, according to protocols for euthanasia in experimental animals [44,45]. After death, the intestinal segment was removed for measurements of length and inside diameter.

The net water flux in the in situ perfusion studies (water absorption and efflux in the intestinal segment) was determined by gravimetric method. The effective absorption rate constant (K_a_) and effective apparent permeability (P_app_) of compounds in the intestinal segments were calculated according to the following mathematical expression [46]:

(1) $$K_{a}=(1-\frac{C_{\mathrm{out}}Q_{\mathrm{out}}}{C_{\mathrm{in}}Q_{\mathrm{in}}})\frac{Q}{V}$$

(2) $$P_{\mathrm{app}}=\frac{-\mathrm{Qln}(C_{\mathrm{out}}Q_{\mathrm{out}}/C_{\mathrm{in}}Q_{\mathrm{in}})}{2\pi \mathrm{rl}}$$

where C_in_ and C_out_ are the concentration of test drug in the effluent perfusate through the inlet and outlet tube (μg·mL^−1^), respectively. Q_in_ and Q_out_ represent the inlet and outlet volume of effluent perfusate (mL), respectively. Q is the volume flow rate of perfusion (mL·h^−1^). V is the volume of the intestinal segment concerned (mL). 2πrl is the area of the mass transfer surface of intestinal segment concerned (cm^2^).

### 4.9. Study for the Absorption of MDQ-TS and Triterpenoid Saponin Monomers in Different Intestinal Segments

To investigate whether the intestinal absorption of MDQ-TS and the six saponins monomers (C1, C2, C3, C4, DC1, and DC2) exhibited segment-dependence and the best absorption site, SPIP was performed in three isolated intestinal segments (duodenum, jejunum, and ileum) with 2.5 mg·mL^−1^ of MDQ-TS, and the equivalent concentration of C1, C2, C3, C4, DC1, and DC2 monomers, respectively.

### 4.10. Study for the Absorption of MDQ-TS at Different Concentrations

To test whether the intestinal mucosa permeability of MDQ-TS exhibited concentration dependent variation, perfusion solutions of MDQ-TS at three concentration levels were separately introduced into the duodenum segment (~10 cm).

### 4.11. Effects of Absorption Enhancers and Membrane Proteins Inhibitors on Absorption of MDQ-TS

K-R buffer solutions with verapamil (200 μmol·L^−1^, P-gp inhibitor), amantadine (2.5 mmol·L^−1^, endocytosis inhibitor) and EDTA (2.5 mmol·L^−1^, absorption enhancers) were separately researched on the intestinal absorptive characteristics of MDQ-TS in situ SPIP of rats to evaluate their effects on MDQ-TS absorption. The rat duodenum (~10 cm) was used to evaluate the permeability in the presence or absence of these agents comparatively.

### 4.12. Studies of the Apparent Oil-Water Partition Coefficient

The equilibrium partitioning of C1, C2, C3, C4, DC1, and DC2 between organic lipid and water (K_ow_) was determined in our study by the traditional shake-flask method (SFM) [18,47]. Briefly, 0.5 mL of each saponin standard working solution (100 µg·mL^−1^ in water-saturated *n*-octanol) was accurately measured into 5 mL centrifuge tubes, and spiked with 0.5 mL of different phosphate buffer solutions (pH: 1.2, 2.0, 3.6, 5.8, 6.8, 7.4, 7.8, and 9.0), respectively. After vortex-mixing for 45 min, the tubes were kept in a temperature-stable horizontal shaking bath (37 °C) for 24 h, and then centrifuged at 13,000 rpm for 10 min. Two hundred microliters of organic supernatant solution was measured, diluted with 800 µL of methanol, and mixed well. Ten microliters of the final solution was injected into the HPLC system (detailed experimental conditions and method validation results were not shown) for determination of the drug concentration in the *n*-octanol phase. The K_ow_ values of the six saponins were calculated according to the following mathematical expression:

(3) $$K_{\mathrm{ow}}=\log\frac{\mathrm{Co}}{\mathrm{Ct}-\mathrm{Co}}$$

where Ct is the total concentration of the drug, and Co is the concentration of the drug in *n*-octanol phase.

### 4.13. Statistical Analysis

All experiments contained five rats per perfusion group. The results were presented as mean ± SD. To facilitate a comparison between each test group versus that of control, statistical analysis was performed using one-way ANOVA with Dunnett’s test by SPSS version 13.0 (SPSS Inc., Chicago, IL, USA). *p* < 0.05 and *p* < 0.01 were considered as marginally and dramatically significant, respectively.

## 5. Conclusions

TCMs have been attracting the attention of scientists as a result of its long-time clinical efficacy and reliable therapeutic actions. However, there has been little information about pharmacokinetic research of Radix *Ilicis Pubescentis* due to the scarcity of the reference substances. The main active constituents of the herb are pentacyclic triterpenoids and their saponins. By now, most pharmacokinetic studies have focused on the single triterpenoid of ilexgenin A, rather than the total triterpenoid saponins and multi-components. In this study, absorption characteristics different from those of monomers were observed in the MDQ-TS administration group. Although the mechanisms of enhanced absorption have not been elucidated, it is quite sure that co-existing ingredients contained in MDQ-TS play an important role in promoting absorption. It would be beneficial to explain the mechanism of the pharmacological effect for improving TCMs. The stronger intestinal absorption of MDQ-TS reflected a holistic and specific view of the compatibility of TCMs. Furthermore, our research on the absorption characteristics of MDQ-TP would also provide references for formulation development, prescription and preparation technology optimization, clinical rational drug use, compatibility mechanism of the herbal medicine, and so on.

## Figures and Tables

**Table 1:** P_app_ and K_a_ of C1, C2, C3, C4, DC1, and DC2 obtained from in situ single-pass perfusion administrated in their monomer forms (*n* = 5).

Intestine	C1		C2		C3	
	P_app_	K_a_	P_app_	K_a_	P_app_	K_a_
duodenum	0.33 ± 0.04	1.77 ± 0.17	0.24 ± 0.01	1.40 ± 0.29	0.23 ± 0.02	1.49 ± 0.36
jejunum	0.35 ± 0.04	1.65 ± 0.19	0.25 ± 0.01	1.72 ± 0.16	0.23 ± 0.05	1.56 ± 0.30
ileum	0.30 ± 0.02	1.50 ± 0.25	0.25 ± 0.03	1.62 ± 0.32	0.21 ± 0.06	1.38 ± 0.06
	**C4**		**DC1**		**DC2**	
duodenum	-	-	-	-	-	-
jejunum	-	-	-	-	-	-
ileum	-	-	-	-	-	-

-: no absorption; P_app_ value units expressed as (×10^−2^ cm·min^−1^); Ka value units expressed as ×10^−2^ min^−1^.

**Table 2:** P_app_ and K_a_ of C1, C2, C3, C4, DC1, and DC2 obtained from in situ single-pass perfusion administrated in MDQ-TS form (*n* = 5).

C (mg·mL^−1^)	Intestine	C1		C2		C3	
		P_app_	K_a_	P_app_	K_a_	P_app_	K_a_
1.25	duodenum	0.22 ± 0.10	1.86 ± 0.73	0.17 ± 0.07	1.10 ± 0.40	0.24 ± 0.10	1.16 ± 0.26
	jejunum	0.17 ± 0.03	0.95 ± 0.28	0.17 ± 0.09	0.74 ± 0.25	0.17 ± 0.04	0.94 ± 0.12
	ileum	0.15 ± 0.03	0.89 ± 0.08	0.14 ± 0.09	1.61 ± 0.37	0.18 ± 0.01	1.04 ± 0.11
2.5	duodenum	0.30 ± 0.09	1.70 ± 0.31	0.27 ± 0.06	0.85 ± 0.20	0.36 ± 0.06	2.19 ± 0.52
	jejunum	0.33 ± 0.08	2.23 ± 0.39	0.26 ± 0.09	0.68 ± 0.18	0.40 ± 0.13	3.82 ± 0.49
	ileum	0.41 ± 0.06	2.90 ± 0.37	0.28 ± 0.04	1.89 ± 0.35	0.42 ± 0.03	3.77 ± 0.41
5.0	duodenum	0.34 ± 0.18	1.68 ± 0.74	0.22 ± 0.13	1.13 ± 0.62	0.39 ± 0.24	1.80 ± 0.97
	jejunum	0.37 ± 0.19	2.30 ± 1.24	0.20 ± 0.13	1.21 ± 0.51	0.40 ± 0.24	2.48 ± 0.86
	ileum	0.25 ± 0.12	1.48 ± 0.76	0.14 ± 0.06	0.85 ± 0.27	0.29 ± 0.15	1.66 ± 0.95
		**C4**		**DC1**		**DC2**	
1.25	duodenum	0.20 ± 0.09	1.04 ± 0.31	0.13 ± 0.05	0.56 ± 0.17	-	-
	jejunum	0.14 ± 0.05	0.84 ± 0.47	0.23 ± 0.15	0.46 ± 0.21	-	-
	ileum	0.14 ± 0.04	0.83 ± 0.28	0.22 ± 0.13	0.59 ± 0.35	-	-
2.5	duodenum	0.42 ± 0.05	1.94 ± 0.50	0.34 ± 0.08	1.76 ± 0.44	0.16 ± 0.06	0.62 ± 0.13
	jejunum	0.49 ± 0.03	3.19 ± 0.56	0.35 ± 0.08	1.99 ± 0.49	0.21 ± 0.08	1.23 ± 0.46
	ileum	0.40 ± 0.03	2.53 ± 0.29	0.16 ± 0.07	1.65 ± 0.33	0.19 ± 0.08	1.54 ± 0.85
5.0	duodenum	0.40 ± 0.22	1.87 ± 0.86	0.30 ± 0.13	1.53 ± 0.55	0.25 ± 0.16	1.27 ± 0.44
	jejunum	0.45 ± 0.18	1.92 ± 0.61	0.32 ± 0.19	2.05 ± 0.72	0.26 ± 0.17	1.73 ± 0.66
	ileum	0.26 ± 0.15	1.52 ± 0.92	0.21 ± 0.11	1.21 ± 0.58	0.19 ± 0.07	1.14 ± 0.45

-: no absorption; P_app_ value units expressed as (×10^−2^ cm·min^−1^); K_a_ value units expressed as ×10^−2^ min^−1^.
